# Contextual Factors That May Impact on the Development and Implementation of the Sugary Drinks Policy: Comment on "Understanding Marketing Responses to a Tax on Sugary Drinks: A Qualitative Interview Study in the United Kingdom, 2019"

**DOI:** 10.34172/ijhpm.2023.7713

**Published:** 2023-03-05

**Authors:** Elisa Chilet-Rosell, Blanca Lumbreras

**Affiliations:** ^1^Department of Public Health, Faculty of Medicine, Universidad Miguel Hernández, Alicante, Spain; ^2^CIBER de Epidemiología y Salud Pública (CIBERESP), Madrid, Spain

**Keywords:** Public Acceptance, Mass Media, Corporate Activities

## Abstract

Forde et al proposed an interesting framework to understand marketing response to a tax in sugary drinks based on stakeholder interviews. Sugary drinks regulation can lead to various strategies in the industry’s marketing activity. That is, it can either result in the industry introducing no changes or it can lead to changes, which can conflict or align with public health objectives. The importance of Forde and colleagues’ analysis lies in the potential for governments to anticipate the industry’s reaction to the legislation and the need of drivers to enable both big and small companies to follow the rules. Governments must not forget the importance of other contextual factors that will have an impact both on the development and implementation of this type of policies and on possible responses that could mitigate their impact such as public acceptance, the influence of mass media and corporate activities aimed at influencing policy.

## Introduction

 In response to the prevalence of overweight and obesity among children and teenagers, one of the measures highlighted by the World Health Organization (WHO) was to increase both the price and taxation of sugary drinks with the aim of reducing their consumption.^1^ A previous systematic review^2^ described that the introduction of this taxation was associated with a higher probability of the purchase of healthy beverages rather than sugary drinks, particularly if this taxation was higher than 20%. This taxation has already been implemented in more than 20 countries worldwide, and in March 2016 the UK government announced the UK Soft Drink Industry Levy (SDIL)^3^ which was implemented in April 2018.

 Studies on the impact of taxes tend to focus on behavioural change on the part of consumers. Forde et al,^4^ however, carried out a qualitative study to explore the responses from sugary drink companies to SDIL. With this aim, they carried out semi-structured interviews with 6 representatives from industry, 6 from academia, and 6 civil society with experience of the strategic decision-making or marketing of sugary drinks companies. Some concerns are related with the methodological characteristics of the study such as the small sample size included (18 representatives, 6 from each category), which could have affected the results. In addition, results from representatives from industry could not fully reflected the responses of the different types of industries following the SDIL (out of the 70 representatives from industry who were initially contacted, only 6 were finally interviewed).

 Considering these limitations, the results of this study showed that interviewees stated that the sugary drinks regulation can lead to various strategies in the industry’s marketing activity. That is, it can either have no impact which results in the industry introducing no changes or it can lead to changes in marketing activity prompted by the impact on sales, which can conflict or align with public health objectives.

 The main conclusion was that companies accelerated and changed their marketing in response to the law through several initiatives such as reformulation, brand acquisition, and changes in packaging, among others. Reformulation was one of the main actions implemented to reduce sugar content and thus avoid paying the levy, mainly in those companies selling products with high- and low-sugar content. A previous analysis evaluated the change in sales after the implementation of SIDL,^5^ that suggested that the amount of sugar purchased in soft drinks decreased by about 30% between 2015 and 2018. The most significant fall in sales occurred between 2017 and 2018, when the law was implemented. According to this analysis, out of the top ten companies, six reformulated most of the products in their brand, and the sales volume increased despite these changes.^6^ Therefore, improvements in public health can be consistent with business success. Nevertheless, there were some barriers to this initiative such as the strength of the brand – which was related to the taste perceived by the consumer – and the great financial effort involved in reformulation which led to small companies being unable to implement it.^4^ In contrast, consumer preference for a healthier product facilitated reformulation towards a product with less sugar. Similar barriers and facilitators influenced other measures such as developing or acquiring new products.

 Forde and colleagues’ analysis suggested that some drivers could be incorporated in the sugar taxation regulation to enable both big and small companies to follow the rules. The authors designed a framework taking into account all the initiatives – and their associated barriers and facilitators- carried out by the soft drink companies in the process of marketing decision-making after the publication of a law such as SDIL. This framework could be considered a relevant tool in the prediction of company-level marketing initiatives after the publication of legislation of this type. The relevance of Forde and colleagues’^4^ proposal lies in the potential for governments to anticipate the industry’s reaction to the legislation and even to limit those changes that can reduce the impact on population health. Furthermore, it supports the need for a comprehensive, multi-pronged approach to regulation aimed at reducing the consumption of sugary drinks. Nevertheless, the article should have explored the tobacco companies’ long-term policy influence strategies to identify lessons to be applied to the sugary drinks regulation. In addition, the study should have made a wider effort to address the commercial determinants of health is crucial to future research and advocacy efforts to challenge of diminish the power of the industry.

 In line with Forde and colleagues’proposal, we consider that governments should include other contextual factors when designing and implementing a levy.

## Public Acceptability

 Beliefs about effectiveness, appropriateness and public trust in industry, government and public health initiatives have important implications in the acceptability of sugary drinks taxation. Another study evaluated differences in the volume and amount of sugar in household purchase of drinks,^7^ showing that the reduction of volume of sugar resulting from a lower purchase of drinks affected by the levy was offset due to an increase in the purchase of untaxed sugary drinks. This could be explained by both the reformulation of drinks from the lower levy to no-levy tier with removal of some but not all sugar, and changes in consumer attitudes and beliefs. A previous survey^8^ on the public acceptability of SDIL found a high support among UK adults although this decreased slightly after its implementation in 2018. A previous review suggested that commitments to hypothecation (earmarking the revenue from health taxes for specific purposes, such as funding health system improvement or obesity prevention), can increase public and political support for taxes.^9^ Greater efforts to support population understanding on public health interventions are required and mass media could have a relevant role in the public’s acceptance.

## Mass Media Influence

 Mass media were predominantly favourable towards taxation as a solution after the publication of Public Health England’s report on sugar reduction^10^ and the publication of evidence of the effectiveness of the Mexican sugar tax.^11^ According to a previous review of the articles published between April 2015 to November 2016, although many studies underlined individual responsibility in the choice of healthier drinks,^12^ the majority pointed to the problem as driven by industrial and environmental phenomena. However, this positive reaction changed after the UK Government’s SDIL announcement, in a similar way to patterns of opposition observed in tobacco control debates.^13^ Given that most of the barriers and facilitators described in the article by Forde et al^4^ suggested the relevant role of the final consumer in the initiatives developed by sugary drink companies, support by the mass media could be key in the implementation of new laws such as SIDL. Mass media are a key partner for policy changes to offset the impact of negative messages.

## Corporate Political Activity

 Aware of the media’s influence on policy changes, sugary drinks corporations engage in media campaigns to ensure the strong presence of Industry positions in the media. These campaigns include the dissemination of information challenging the need or timing for legislation on sugar-sweetened beverage taxes. In the case of Ireland, a company-funded report provided evidence in favour of reformulation and questioned the need for further legislation.^14^

 There is both theoretical and empirical literature describing companies’ activities and warning that one of their aims is to preserve and maximise profits through a variety of strategies to influence policy including blocking public health protection policies.^15^ It is not only the media that are the target of these corporate strategies, but also and particularly governments. A recent paper by Lauber et al^14^ analysing the experiences of key stakeholders with regard to the sugary drinks tax policy found that they all observed interference by commercial actors in reference to this policy. Interviewees described different strategies that include information management — including that of the media —, direct participation and access to the policy process; and questioning the legality of the proposed legislation at national or international level. Although perhaps the most paradigmatic case is that of the tobacco industry, the food industry also shapes science, by funding and disseminating research and information to serve its interests, and criticises evidence that may frustrate these interests.^16-19^

## Conclusion

 Forde et al proposed that legislation has an impact on decisions in the sugary drinks industry, some of which are positive such as reformulation, but these changes depend on both company-specific and contextual factors. Governments should incorporate drivers in the sugar taxation regulation to enable companies, regardless of their characteristics, to contribute to public health. The framework proposed by Forde et al allows policy-makers to consider companies’ possible reactions to public health legislation affecting their products. In the process of developing these policies, governments should consider the importance of other contextual factors that will have an impact both on their development and implementation and on possible responses that could mitigate their impact such as public acceptance, the influence of mass media and corporate activities aimed at influencing policy.

## Ethical issues

 Not applicable.

## Competing interests

 Authors declare that they have no competing interests.

## Authors’ contributions

 Both authors contributed to the conception, design and drafting of the commentary, and critical revision of the manuscript for important intellectual content.
